# 

**Published:** 1867-12

**Authors:** 


### THE CHICAGO MEDICAL JOURNAL.

Edited by J. ADAMS ALLEN, M.D., LL.D.
Prof. Principles and Practice of Medicine and Clinical Medicine in Rush Med. College.

All letters for the JOURNAL should be addressed to DR. J. ADAMS ALLEN, P.O. Box 1948, Chicago, Ill.

**TERMS---$2 PER ANNUM, IN ADVANCE.**

$3 if not paid before 1st of July in each year. Single copies, 25 cents.

---

### CONTENTS FOR DECEMBER.

International Medical Congress,---------- 561

Case Supposed to be Carcinoma of the Stomach. By M. A. McClelland, M.D., Knoxville, Ill.,---------- 593

Case of Fibrous Tumor Removed from the Broad Ligament of Uterus. By Walter Burnham, M.D., Lowell, Mass. Reported by Chas. M. Clark, M.D., Chicago, Ill.,---------- 596

Dr. Burnham's Report of the above Operation.---------- 599

Shell Wound in the Forehead. By A. A. Dunn, M.D.,---------- 601

Conservative Surgery. By C. F. Hart, M.D.,---------- 604

Chicago Medical Society,---------- 606

Dangerous Effects of Chloroform. Chronic Asthma Relieved by Electricity. Rupture of the Spleen. Singular Fracture of the Os Calcis. Retained Placenta. Stricture, Enlarged Cancerous Liver. Death from large doses of Digitalis. Bending of the Radius and Ulna. Perforating Ulcer of the Ileum. Facial Paralysis in Syphilis. Tetrachloride of Carbon. Bromide of Potash in Epilepsy. Valvular Disease of the Heart,---------- 606

CORRESPONDENCE.

EDITORIAL.

The December No. of the Journal for 1867,---------- 618

Professorial Changes,---------- 619

Nil Admirari,---------- 620

A Voice from Lilliput,---------- 621

Obituary,---------- 622

Note from Prof. Rea,---------- 622

Change of Base,---------- 623

Receipts Acknowledged,---------- 623

To the Medical Profession,---------- 623

Winter Retreat for Consumptives,---------- 623

### INDEX.

PAGE.

Appointment, ---------------- 139
American Medical Association, -- 143
A New Form of Suture,--------- 150
Abortion, Criminal, ------------ 221
Apropos of the Endoscope,------ 232
Advertisement. ---------------- 238
Atomizing Instruments,--------- 245
Anterior Dislocation of the Elbow-Joint, --------------------- 251
Apologetic,-------------------- 263
American Naturalist,----------- 263
American Medical Association,-- 264
American Pharmaceutical Ass'n,- 265
Astronomical, ----------------- 333
Asthma, Treatment of,---------- 367
A New Form of Suture,--------- 373
Alumni Meeting, -------------- 380
A Chapter about Books,------- - 384
A Paper read before the Chicago Medical Society,------------- 532
Allen County, Ind., Med. Soc'y,- 329
Asthma, Chronic, relieved by Electricity,------------------ 607
A Voice from Lilliput,---------- 621

Bending of the Radius and Ulna, 609
Books Received, --------------- 616
Bromide of Potash in Epilepsy,-- 610
Book Notices,------------41, 136, 262
Books Received,--------226, 480, 543
Booksellers, W. B. Keen & Co.,-- 486
Board of Health, Chicago,------- 217
Books and Pamphlets Received,- 446
Belladonna, Scarlatina,--------- 232
Back No.'s of the Journal Want'd, 238
Bellevue Place,----------------- 334

Carbon, Tetrachloride of,-------- 609
California Wines, -------------- 486
Carbolic Acid,------------------ 469
Conservative Surgery,---------- 604
Cancerous Liver, Enlarged, ----- 608
Carcinoma of the Stomach,------ 593
Cerebro-Spinal Maningitis. By Dr. Clark,------------------- 1

PAGE.

City Mortality for Dec., 1866,---- 47
" " Jan., 1867,---- 96
Cliniques at Rush Med. College,- 543
Consanguineous Marriages,------ 488
Chicago Medical Society,-------- 473
Connubimur. By T. O. Edwards, M.D.,---------------------- 56
Cholera, Treatment of,---------- 60
Consumption, Curability of, ----- 77
Chicago Medical Society,-------- 83
Cerebro-Spinal Meningitis, Fifteen Cases, --------------------- 104
Chicago College of Pharmacy,--- 142
Clinical Lectures. By W. C. Lyman, M.D.,------------------ 153
Criminal Abortion,-------------- 221
Cholera, ----------------------- 228
Calculus, Vesical, -------------- 228
Clitoridectomy, ---------------- 228
Cincinnati Again,--------------- 223
Copyright, --------------------- 225
Committee on Practical Medicine and Epidemics, ------------- 237
Chicago Medical Society,-------- 258
Cancer, Treatment of,---------- 269
Chicago Medical Society,-------- 606
Chicago Medical Society,-------- 305
Cancerous Disease of the Stomach and Liver, ----------------- 305
Communications,--------------- 332
Contagion of Cholera,----------- 355
Contagiousness of Cholera,------ 377
Canada, ----------------------- 379
Cancerous Disease of the Œsophagus, with Ulceration into the Bronchia,------------------ 433
Curvature of the Spine,--------- 434
Cincinnati Changes,------------ 444
Crowded out,------------------- 446
Chloroform, Dangerous Effects of,- 606
Chronic Asthma relieved by Electricity,--------------------- 607
Correspondence. By H. O. Hitchcock, M.D., Kalamazoo, Mich.,- 615

PAGE.
Correspondence. By J. B. Fares, M.D., Romeo, Mich., ---------- 614
Correspondence. By E. R. Hutchins, M.D., Philadelphia, -------- 612

Digitalis, Death from large doses, 609
Dangerous Effects of Chloroform, - 606
December No. of the Journal, 1867, 618
Death from large doses of Digitalis 609
Dislocations of the Shoulder, Reduction by Manipulation. By D. G. Rush, M.D., of Chicago, - 49
Deformities of the Nose, --------- 71
Disease of Tricuspid Valves, ------ 84
Death from Hemorrhage, -------- 112
Dr. Brown's Lecture before the Macon Co. Medical Society, --- 125
Dr. Lewitt, -------------------- 234
Dry Tongue, ------------------ 231
Dislocation of the Elbow-Joint, Anterior, ------------------ 251
Delay, ------------------332, 482
Death of Dr. Hunt, ------------- 384
Deaths of Noted Physicians, ----- 485
Dr. Carleman's Portable Russian Vapor Bath Apparatus, -------- 486
Death of Dr. Orrin Smith, ------- 487

Electricity, Chronic Asthma relieved by, ------------------ 607
Enlarged Cancerous Liver, ------ 608
Eye, Introduction to the Course of Lectures on Diseases of. By E. L. Holmes, M.D., Surgeon to the Charitable Eye and Ear Infirmary, etc., --------------- 11
Editorial, -----------41, 86, 134, 209
Errata, ------------------------ 48
Extract from a Paris Letter, ----- 81
Extirpation of Thyroid Gland, --- 97
Editor's Salutation, ------------ 134
Endoscope and its Application to the Diagnosis and Treatment of Urinary Affections, ----------- 177, 273, 385, 378, 449, 545
Exchanges and Correspondents, -- 209
Elimination in Cholera, --------- 229
Erratum, ---------------------- 237
Epilepsy treated by Lactate of Zinc, ------------------------ 299
Eye, Glaucoma, ---------------- 305
Enlargement of the Colon. By W. Lewitt, M.D., ------------ 359
Electro-Magnetism, ------------ 371
Erratum, ---------------------- 377
Encephaloid Mass Penetrating the Right Renal Vein and the Vena Cava, ----------------- 438

PAGE.
Erratum, ---------------------- 446
Endoscope, -------------------- 337
Extracts from a Private Letter from Prof. Freer, ------------ 538
Epilepsy, Bromide of Potash in, - 610

Fracture of the Hip-Joint treated without Splints, ------------ 72
Fracture of Astragalus, Dislocation of Foot Inwards, -------- 252
Fox River Valley Med. Ass'n, --- 174
Fibroid Polypus of the Uterus, -- 305
Fatty Degeneration of the Kidneys in Phthisis, ------------ 473
Free Dispensary, --------------- 482
Fibrous Polypus of Uterus, Removal by Ligature, ------------ 513
Fibrous Tumor, Removal of, ----- 596
" " Dr. Burnham's report of operat'n 599
Facial Paralysis in Syphilis, ----- 609
Forehead, Shell Wound in, ------ 601

Gayetty Outdone, -------------- 332
Gelseminum, ------------------ 375
Gelsemium, -------------------- 610
George K. Amerman, M.D., ----- 444

Hospital, Proposed, on Randall's Island, New York, ----------- 96
Hemorrhage, Death from, ------- 112
Hydrophobia, ----------------- 231
Homœopathists *vs.* Homœopathy, 268
Hæmorrhoidal Affections, ------- 334
Headland on the Action of Medicines, ----------------------- 487
Heart, Valvular Disease of the, -- 610

Iowa, Medical Aspect of, -------- 114
Iowa and Illinois Central District Medical Association, --------- 124
International Medical Congress at Paris, ---------------235, 236, 561
Illinois State Medical Society, --- 236
Indiana State Medical Society, --- 236
Incontinence of Urine successfully treated by Ext. of Belladonna, 271
Illinois State Medical Society, --- 308
Indiana, Medical Association, Allen County, ------------------ 329
Inhalations of Atomized Fluids, - 420
Intraocular Tumor, ------------ 475
Ileum, Perforating Ulcer of the, -- 609

Journal, Dec. No. of, 1867, ------ 618
Laryngeal Tumor, ------------- 84
Lewitt, Dr., Complimentary Letter to, --------------------- 233
Light for the Endoscope, -------- 231

PAGE.
Large Concretions nearly filling the Globe, ---------- 306
Laying the Corner-Stone of Rush Medical College, ---------- 401
Liver, Enlarged Cancerous, ---------- 608

Medical Societies, Proceedings of, 19
Military Tract Association, ---------- 26
Medical Society, Esculapian, ---------- 29
" " Morgan Co., --31, 122
" " Chicago, ---------- 83
" " Kalamazoo Valley, Mich., ---------- 176
" " Illinois State, ---------- 236
" " Indiana State, ---------- 236
" " Chicago, ---------- 258
" " Illinois State, ---------- 308
" " Chicago, 432, 473, 606
" Societies, ---------- 377
Malarial Disease, Treatment of, ---------- 53
Menorrhagia, Climacteric, ---------- 65
Microscope. By Professor O. W. Holmes, ---------- 74
Morgue in New Orleans, ---------- 80
Medical Aspect of Iowa, ---------- 114
Morgan County Medical Society, ---------- 122
Milk-Sickness and Illinois Legisture, ---------- 163
Milk-Sickness, ---------- 229
Medical Bookstore, ---------- 235
Meeting of the Societies, ---------- 235
Married, ---------- 237
Mortality Report for February, ---------- 239
" " March, ---------- 240
" " May, ---------- 336
Milk Sickness, ---------- 253
Military Tract Medical Assoc., ---------- 326
Married, ---------- 335
Meeting of the Alumni of Rush Medical College, ---------- 443
Medical Books, ---------- 484
Modern Anæsthesia, ---------- 487
Married, ---------- 488

New Medical Journal, ---------- 234
Nasal Polypus, ---------- 429
Neuralgia produced by a Fatty Tumor, ---------- 473
New Medical Journals, ---------- 484
New Uterine Supporter, ---------- 486
Nil Admirari, ---------- 620
Note from Prof. Rea, ---------- 622

Obituary, ---------- 238
Ovariotomy, ---------- 241
Obituary of Dr. G. K. Amerman, ---------- 384
Opium and Belladonna, ---------- 529

PAGE.
Ovarian Tumor, Successful Removal of, ---------- 434
Os Calcis, Singular Fracture of, ---------- 608
Obituary, ---------- 622

Prolapsus of the Rectum, ---------- 145
Puerperal Convulsions, Testimony in favor of Belladonna, ---------- 161
Puerperal Convulsions controlled by Inhalation of Chloroform, ---------- 155
Phthisis Pulmonalis as a Sequel to Suppressed Menstruation, ---------- 171
Poisoning by Strychnia, ---------- 229
Prof. M. Gunn, ---------- 234
Pass him around, ---------- 224
Propolis as a Remedy for Diarrhœas, Acute or Chronic, ---------- 417
Personal, ---------- 484
Propolis, ---------- 485
Parasite, Singular, ---------- 83
Pulaski County, Ind., Med. Soc'y, 175
Practical Anatomy, ---------- 213
Progressive Paralysis of the Insane, ---------- 365
Peoria County Medical Society, ---------- 435
Physiological and Therapeutical Action of Cod-Liver Oil, ---------- 556
Placenta, Retained, ---------- 608
Perforating Ulcer of the Ileum, ---------- 609
Paralysis, Facial, in Syphilis, ---------- 609
Potash, Bromide of, in Epilepsy, ---------- 610
Professorial Changes, ---------- 619

Quinine, East India, ---------- 68

Rush Medical College, ----95, 140, 377
" " new build'g, 210
" " corner stone, 334
" " meeting of Alumni, 443
" " Proceedings Intr'd'ct'y, 489
Resignation of Prof. Gunn, ---------- 233
Receipts Acknowledged, ---------- 238, 272, 335, 384, 488, 623
Removal of Uterine Polypus, ---------- 254
Report of Cholera in Chicago, in 1866, ---------- 289
Reducing the Temperature, ---------- 333
Rabies, ---------- 361
Retarded Congenital Syphilis, ---------- 362
Retention of Urine from Stricture, Perineal Section, Recovery, ---------- 468
Rape; What is It? ---------- 472
Retinitis associated with Bright's Disease, ---------- 474
Removal of Fibrous Tumor, ---------- 596

PAGE.
Rupture of the Spleen, 607
Retained Placenta, 608
Radius and Ulna, Bending of the, 609

Spinal Cord, Investigations of, in Tetanus, 77
Scabies—Paraffine, 82
Singular Parasite, 83
Sanitary Measures, 85
Settlement of Physicians' acc'ts, 95
Suture, New Form of, 150
Scirrhus of the Liver and Stomach, Tuberculosis and Partial Fatty Degeneration of the Kidneys, 166
Strychnine, Poisoning by, 229
Southern Journal of Med. Science, 234
Supplement, 332
Scintillations of Similia, 333
Selections from Surgical Notes. By Prof. Gunn, 353
Sutures, New Form, 373
St. Louis Good Samaritan Hosp'l, 374
Supplement, 377
Shell Wound of the Head, 432
Spine, Curvature of, 434
Spinal Irritation, 5[illegible]6
Shell Wound in the Forehead, 601
Stomach, Carcinoma of, 593
Spleen, Rupture of the, 607
Singular Fracture of the Os Calcis, 608
Stricture, 608
Syphilis, Facial Paralysis in, 609
Surgery, Conservative, 604

Tricuspid Valves, Disease of, 84
Thyroid Gland, Extirpation of, 97
To Correspondents, 138

PAGE.
Typhlitis, 159
The Chicago Board of Health, 217
To Physicians, 232
To Subscribers, 262
Thanks, 268
The Illinois Homœopathists, 263
The Chicago College of Pharmacy, 264
To the American Medical Ass'n, 266
The Treatment of Cancer, 269
The Ventilating Method, 272
Three Surgical Cases, 3[illegible]1
The Corner Stone of Rush Medical College, 334
To Graduates of Rush Med. Coll., 334
Tumor of the Choroid, 435
To Subscribers, 444
The Relation of the Physician to the Purity of the Articles used in Prescriptions, 465
The College Museum, 483
To Correspondents, 483
The Next Volume, 542
Tetrachloride of Carbon, 609

University of Michigan, 235
" Wisconsin, 378
Ulcer of the Ileum, Perforating, 609

Valedictory, 134
Veratrum Viride, 171
Vesical Calculus, 228
Venomous Bites, 368
Valvular Disease of the Heart, 610

Why Not? 225
Wants to Know, 225
Women Doctors, 443

Yellow Fever, 484

***Receipts Acknowledged from:***

H. F. Livingston, $3; W. M. Sweetland, 3; A. R. Logan, 3; G. Ryan, 7; Geo. Egbert, 5; James Barr, 2; Thos. Fravel, 3; C. M. Smith, 5; J. D. Williams, 2; R. Carscadden, 0.50; Dr. Buckworth, 2; R. Broughton, 3; J. R. Spooner, 1.50; W. F. Green, 3; John H. Goodell, 2; M. R. Fowler, 2; Clark & Seely, 2; Adair & Catlin, 1.50; W. H. Coover, 5; J. M. Damron, 5; D. Rea, 5; J. G. Riddler, 5; John Little, 1; S. H. Whittasey, 2; E. T. Glassner, 6; G. W. Covert, 2; G. W. Kirkpatrick, 5; J. B. Corey, 6; J. W. Garven, 5; A. E. Smith, 3; T. E. Annis, 2; Dr. Kimbell, 3; G. F. Keiper, 2; J. H. Barnwell, 5; W. L. May, 5.

---

TO THE MEDICAL PROFESSION.

Wishing to retire from the practice of medicine, I offer for sale a splendid residence, office, and a practice worth from $2500 to $4000 per annum, situated in the *very wealthiest* part of Iowa. Society, church, and educational privileges all that can be desired. *Village and Country Practice.*

*Terms:*—$3000. $1700 cash down, balance secured by the property, and to draw interest at the rate of 10 per cent till paid. Address DR. KITTLE,

Box No. 2. *Marshall, Henry Co.. Iowa.*

